# Determinants of Face Recognition: The Role of Target Prevalence and Similarity

**DOI:** 10.5334/joc.339

**Published:** 2024-02-21

**Authors:** Lionel Boudry, Jeffrey D. Nador, Meike Ramon

**Affiliations:** 1 Applied Face Cognition Lab, University of Lausanne, Lausanne, Switzerland

**Keywords:** Face identity processing, Face recognition, Target Prevalence, Expectation/context

## Abstract

Studies of facial identity processing typically assess perception (via matching) and/or memory (via recognition), with experimental designs differing with respect to one important aspect: Target Prevalence. Some designs include “target absent” (TA) among “target present” (TP) trials. In visual search tasks, TA trials shift an observer’s decisional criterion towards a stricter one, increasing misses. However, decisional biases will differ between individuals and across an individual’s decisions as well. In this way, excluding TA trials ensures comparable levels of expectation and thus a more controlled decisional bias both within and between observers by not considering correct rejections and false alarms. However, TA trials may occur, e.g., in police line-ups, where it is important to consider observers’ face recognition ability net of the potential biases introduced by TA and TP trials. And, while these have been investigated in numerous other stimulus domains, their effects have not yet been extended to face recognition. We therefore sought to fill this void by testing different versions of the previously established Models Memory Test, which measures old/new recognition of experimentally learned facial identities. Our study found significant expectation effects, driven by target prevalence that persist even given prevalence changes. This implies that face recognition – even measured with naturalistic changes – is influenced by prior perceptual decisions.

## Introduction

Over the past two decades, scientific interest in face identity research has increased dramatically. Searching the term “Face Processing” on pubmed.com yields *267* articles published in the year 2000. Now, twenty years on, the same search returns seven times this number. Several technological advances facilitating facial image creation and processing have contributed to this growth, including proliferation of devices with cameras, alongside rapid improvements in machine learning and artificial intelligence (AI) algorithms. These developments have largely been benchmarked using instances where accurate processing of facial identity is paramount, as in security and law enforcement settings (Ramon et al., 2019; Ramon & Rjosk, 2022; Mayer & Ramon, 2023).

Assessing the extent of the benefits that these technological advances can provide requires thorough knowledge of human performance as a benchmark. Therefore, psychological studies over this same period have sought to characterize ability in face identity processing (FIP). These lines of research include neuropsychological studies examining the effects of brain damage (Ramon, Busigny, Gosselin & Rossion, 2016; for reviews see Rossion, 2022 a, b), fundamental research investigating how real-life experience shapes measured FIP (for reviews see Ramon & Gobbini, 2018; Meissner & Brigham, 2001), and individual differences among neurotypical individuals (Fysh et al., 2022; Stacchi et al., 2020; Bobak et al., under review). A recent subset of studies have focused on individuals with superior skills, so-called Super-Recognizers (Russell, Duchaine & Nakayama, 2009; Ramon, 2021), to characterize the mechanism(s) underlying their unique ability (Nador et al., 2021 a, b; 2022; Linka et al., 2022) and how to identify them (Mayer & Ramon, 2023; Ramon & Rjosk, 2022; Ramon, 2021). Consequently, there has been a surge in the development of FIP assessment tools, which typically measure specific subprocesses with varied (at times suboptimal) reliability and precision (Fysh & Ramon, 2022; Bobak et al., under review; Stacchi et al., 2020; Fysh et al., 2020).

### Target Prevalence in tests of FIP and visual search

Across professional domains, FIP measures have been developed for several reasons (for review, see Young & Ellis, 1989). For instance, a body of neuroscientific research aims to understand FIP’s subprocesses and neural correlates (Rossion et al., 2020; Yovel, 2016) In law enforcement, understanding FIP differences is important e.g., in the context of perpetrator identification through testimony of witnesses or forensic professionals (Mayer & Ramon, 2023). The motivation for studying FIP typically influences a range of methodological choices. These can relate to performance measures considered, e.g., accuracy or response time for identity matching (see. Fysh & Ramon, 2022; Nador et al., 2022), or experimental design. While some studies seek to maximize ecological validity (e.g. Bate et al., 2018) by applying natural and realistic changes to stimuli, others may artificially increase task difficulty by adding ambient noise to their stimuli (e.g. Russell et al., 2009).

Furthermore, to approximate real-life scenarios, some studies consider the effect of *Target Prevalence* (the presence of target identities among foils during/across experimental trials) on FIP. Thus, in 1-to-many matching, or *n*-alternative forced-choice recognition tasks, the target identity signal is often *absent* from the possible response options on a subset of trials. (Bruce et al., 1999; Bate et al., 2018). This is thought to serve as a model for myriad real-world scenarios, including policing and security. For example, a mug-shot line-up created by the police may either include the depiction of a person of interest (target present), or not (target absent). Ideally, witnesses and professionals should not only be able to recognize persons of interest (or “targets”) when present, but also refrain from falsely identifying others in the lineup (“foils”) as the perpetrator, whether or not the target is absent. However, to the best of our knowledge, no such studies to date have systematically varied *Target Prevalence* during face recognition tasks, leaving substantial doubt (warranted or not) in witnesses and professionals’ judgments.

This doubt arises from more domain-general work on visual search, wherein the role of *Target Prevalence* is routinely studied in diverse scenarios, such as screening baggage at airport security for weapons (Wolfe & Van Wert, 2010; Wolfe et al., 2007), or screening radiological images to diagnose tumors (Nakashima et al., 2013). Critically, in both fields, targets are exceedingly rare. In mammography, for example, only 3% of scans present a tumor (Gur et al., 2004). In radiology, low *Target Prevalence* has been shown to induce miss rates as high as 30% for tumors after scan examinations (Evans et al., 2013). In airport security, baggage screeners reportedly miss 95% of weapons hidden in luggage (Fishel, Levine, & Date, 2015); some recent estimates hold that only 10 firearms are identified per million passengers screened (Transportation Security Administration, 2015).

Overall, researchers have shown that hit rates – the proportion of correctly identified targets among foils – decline drastically when targets are rare (Wolfe et al., 2007). This “low prevalence effect” is a major concern for visual search tasks in general, presumably also including FIP. Importantly, though, this effect arises due to observers’ inherent bias towards signaling the presence or absence of a target, such that when targets are rarer, observers are less likely to signal their presence. It should be noted, though, that this need not necessarily imply decreased sensitivity to targets; observers also make fewer false alarms (incorrectly identifying a foil as a target) under such circumstances (Wolfe et al., 2007; Peltier & Becker, 2016).

### Effects of Target Prevalence and Similarity on face memory

In practice, FIP-related tasks often – but not always – require *memory* of a given facial identity. On one hand, for instance, police officers may screen CCTV footage for the presence of a particular suspect whose photograph they have in hand. On the other, a witness may need to identify a suspect specifically from memory. Unfortunately, false alarms in these scenarios have serious ramifications, and eyewitness testimony is extremely prone to false alarms, to the point that they are among the most common causes of suspect misidentifications (Wells & Olson, 2003).

Consequently, the inclusion of *Target Absent* trials experimentally has become a priority (e.g. Bate et al., 2018; Bruce et al., 1999; Matthews & Mondloch, 2018), leading researchers to proffer many such assessments. However, no such studies have systematically varied *Target Prevalence* to assess changes in hit rate, and remain prone to bias as they include *Target Absent* trials.

**Figure 1:**
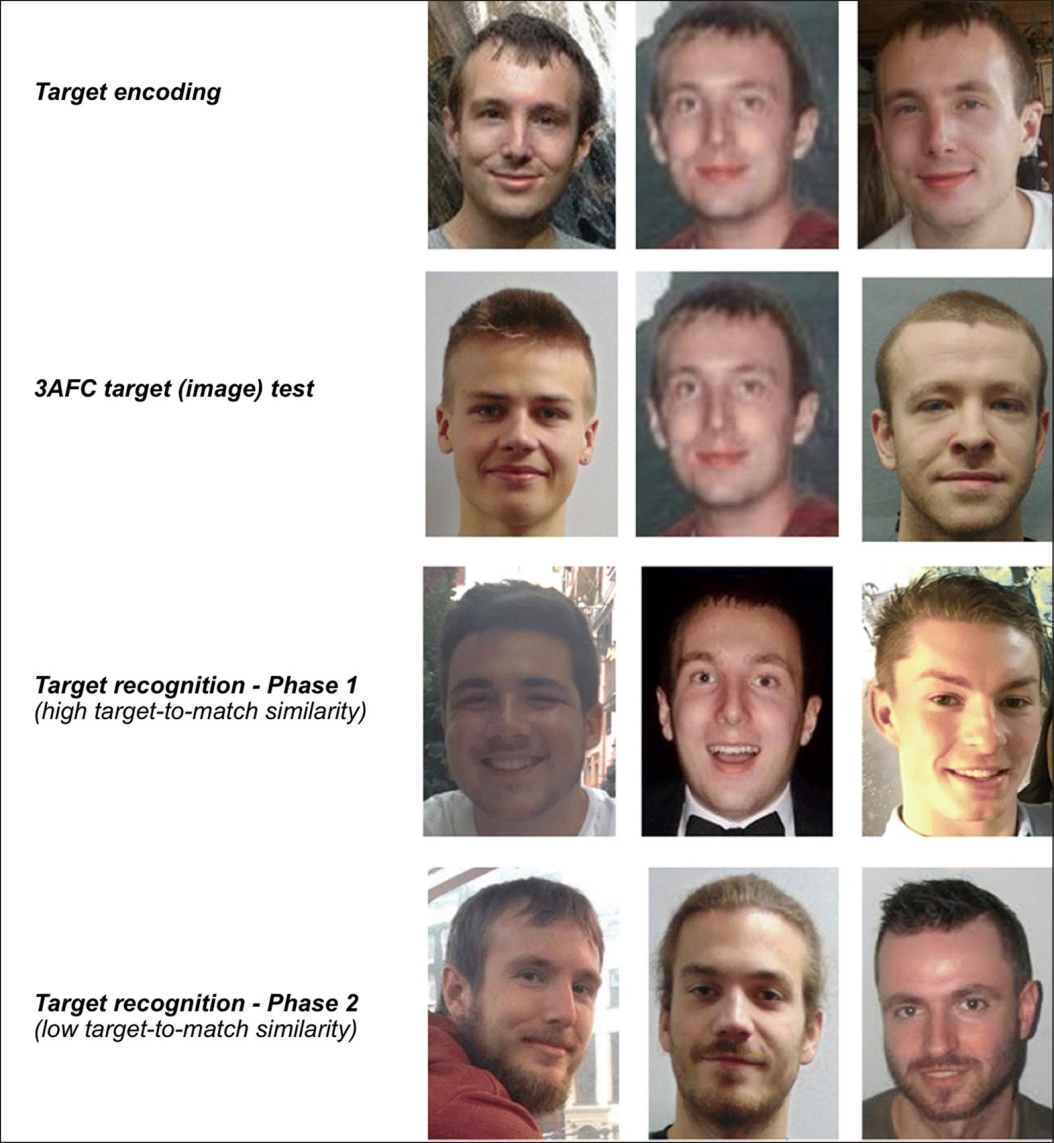
**Examples of stimuli presented in the Model Matching Test across test phases**. Images are reproduced from Bate et al. (2018) under a Creative Commons Licence (http://creativecommons.org/licenses/by/4.0/).

To address this, we adapted one such assessment tool, the Models *Memory Test* (MMT; Bate et al., 2018). The MMT measures recognition performance for learned face identities using “ambient images” (Jenkins & Burton, 2011), i.e. naturally occurring variability in facial appearance. Throughout two target recognition phases, observers are presented with triplets of images containing two distractor and one target identity. Both phases differ in the similarity between initially learned target images and the potential matching target stimulus. Similarity can be high, with minor changes between the learned image of a given identity and its matching probe, or low, i.e. entailing greater changes (see Figure 1). Additionally, the MMT includes *Target Absent* trials at a constant rate of 50% of the trials throughout, and as such cannot assess the effect of varying their prevalence on hit rates. Therefore, we extended it to include conditions with only *Target Present* trials.

Practically, naturally occurring changes in facial appearance negatively impact face recognition (Patterson & Baddeley, 1977). The MMT exploits this effect of image changes to systematically increase *Target-To-Match Similarity*. That is, across the recognition phases, targets’ facial appearance changes are initially less, and then more pronounced across Phases 1 and 2 (see Figure 1). Unfortunately, the parallel implementation of target absent trials as a second novel feature of the original MMT is undesirable. Simply put, differences between high and low similarity conditions could have been explained by either or both of these methodological considerations (*Target Prevalence* or *Similarity*). And, since these factors operate in concert to create a specific *context* for face recognition performance, a lack of simultaneous control over them both limits the original MMT’s insight into face recognition memory performance. Our inclusion of *Target Present-Only* conditions remedies this issue.

### Closing the gap: contextual effects on face recognition

For this study, *Context* comprises previously acquired experience within an ongoing situation. Operationally, this translates to the effect that previous trials (or previous phase) have on processing current stimuli, along with their potential consequences for future stimuli (Zimmermann et al., 2007). In visual search tasks, context is often manipulated via priming, through presentation of targets or foils (Kristjánsson & Campana, 2010). However, visual search studies overlook these “implicit” contexts, wherein a given experience or percept can affect the following one(s). For example, an observer’s previous experience with low *Target Prevalence* could bias their hit rates downwards on subsequent trials. Exploring the effect of *Context* in a visual search task, Wolfe and colleagues (2007) reported that training with higher *Target Prevalence* led to better subsequent performance in low and high *Target Prevalence* visual search tasks.

We reasoned that performance should be facilitated by higher vs. lower similarity between images used during learning and recognition, leading to increased hit rates. Additionally, we hypothesized that hit rates would be reduced when including target absent trials compared to when excluded. A higher *Target Prevalence* would increase recognition performance as discussed earlier in the context of visual search more generally. Finally, we hypothesized that contextual effects of *Target Prevalence* would show carry-over within observers, such that those who were first exposed to low target prevalence would show lower hit rates in future perceptual decisions and vice-versa.

## Methods

All research procedures were approved by the local Ethics Committee (Approval Number 473, University of Fribourg, Switzerland) and conducted following the tenets of the Declaration of Helsinki (Puri, Suresh, Gogtay, & Thatte, 2009).

### Observers

An a priori power analysis determined that at least 14 observers would be necessary to detect medium-sized effects at α = .05 and β = .8 given our experimental design. Invitations for remote participation were sent out to sixty relatives of one experimenter, all of whom participated in the experiment (half female; mean age: 40±12 years), and who had normal or corrected-to-normal vision. The observers were unaware of the study’s purposes concerning *Target Prevalence, Target-to-Match Similarity, Context* and *Cultural Exposure*. Observers were randomly assigned to one of three groups, and each group completed a different version of the MMT (see Table 1). According to their own accounts, observers were exposed to South Asian (SA), Western Caucasian (WC), or ethnically-mixed groups (SAWC) (see below).

**Table 1:** **Demographic information of observers assigned to the three versions of the Models Memory Test (MMT)**. Groups were exposed to different combinations of *Target Prevalence* across Phases 1 and 2, which contained only target present trials (TP), or included target absent trials (TA) (as in Bate et al.’s (2018) original study). For all groups, Phase 1 and Phase 2 were characterized by high and low *Target-to-Match Similarity*, respectively, due to the degree of ambient changes among images across phases. Each group first completed the first, “easier”, followed by the second, “harder” phase (with lesser vs. greater ambient changes). For Group 1, Phase 1 had low *Target Prevalence* (TA/TP; i.e. target absent *and* target present trials) and Phase 2 had high *Target Prevalence* (TP; i.e. only target present trials). This pattern of *Target Prevalance* was reversed in Group 2, where Phase 1 had high (TP), followed by low *Target Prevalance* in Phase 2.


CONTEXT (PHASE1–PHASE2)	CULTURAL EXPOSURE OF PARTICIPANTS ASSIGNED TO CONTEXTS *N* (MALE/FEMALE); AGE ± SD		

	WESTERN CAUCASIAN (WC)	SOUTH ASIAN (SA)	MIXED (SAWC)

Group 1: Low-to-high *Target Prevalence* (TA/TP—TP)	7 (3/4); 35 ± 6	7 (3/4); 45 ± 13	6 (4/2); 41 ± 11

Group 2: High-to-low *Target Prevalence* (TP—TA/TP)	7 (3/4); 40 ± 12	6 (3/3); 43 ± 14	6 (2/4); 34 ± 9

Group 3: Low-to-low Target Prevalence* (TA/TP—TA/TP*)	6 (4/2); 43 ± 18	7 (3/4); 39 ± 12	7 (5/2); 39 ± 11


* NB: This is the original MMT reported by Bate et al. (2018). TA/TP: indicates that a phase contains both target absent (TA) and target present (TP) trials. TP: indicates that a phase contains only target present (TP) trials.

### Inter-Ethnicity Social Contact Questionnaire (IESCQ)

We assessed our observers’ contact with/exposure to different ethnicities to ensure that any such exposure differences would be balanced across groups. To this end, we designed a novel, self-administered *Inter-Ethnicity Social Contact Questionnaire* (IESCQ), which was implemented online beforehand and took between five and ten minutes to complete.

The IESCQ contains 10 closed-ended questions soliciting self-reports of the quality and quantity of own-ethnicity (South Asian or Caucasian; five items) and other-ethnicity (South Asian or Caucasian; five items) exposure and contact. IESQCQ items assess exposure within their work setting, various social/public settings, through personal knowledge, digital media, etc. For example, Item 2 asked, “*Consider your experiences with Caucasians (White people) within the context of various social/public settings. Approximately, what percentage of the people you regularly interact or socialize with are Caucasians?*”. The IESCQ uses the same items for own- and other-ethnicity. All observers rated their response to each item on a percentage scale from 0 to 100 in increments of 10%, 0 being no contact/exposure at all and 100 being maximal, daily contact. Mean percentage scores were calculated for each observer for each of the two ethnicities. Observers with low to no exposure/contact with the other ethnicity (0–30%) or high exposure/contact with their own ethnicity (70–100%) were assigned to a mono-ethnic group, whereas observers with relatively similar exposure/contact to both ethnicities were placed in the multi-ethnic group.

### Stimuli and General Procedure

All experimental stimuli were taken from the original MMT (Bate et al., 2018). They depict naturalistic, full-color, adult male faces taken under different lighting conditions and from various viewpoints. Stimuli presented in Phase 2 included additional paraphernalia (greater changes) (e.g., addition of reading glasses, beanies, facial hair, etc.). Target face stimuli included 14 “ambient” images (Jenkins & Burton, 2011) of 6 target identities; foil face stimuli consisted of 300 images, each displaying a different identity. Images preserved all external features of the face including hair and ears.

Each TP trial involved presentation of three probe stimuli: one of a target identity and two foil identities. Each TA trial involved presentation of three probes displaying foil identities. Observers participated online (testable.org), using their personal computers’ web browser of choice, in full-screen mode. Prior to commencing, they were asked to make sure they could avoid distractions and to position themselves at one arm’s length distance from the screen. Comparable on-screen stimulus size was ensured through a default calibration procure.

### Procedures & Different Versions of the MMT Used

The original MMT’s design was delivered to Group 3, where an initial *Target Learning* (*encoding* and *target test*) was followed by two *Recognition Phases* of 45 trials each with low *Target Prevalence* (equal proportion of TP and TA trials in each *Recognition Phase*). Recognition phases differed in terms of *Target-To-Match Similarity* (i.e., similarity between the learned targets and probes presented during recognition phases). As demonstrated in Figure 1, Phases 1 and 2 involved lesser vs. greater changes (change of lighting or viewpoint, vs. change of hairstyle, addition of a beard, glasses, etc), respectively.

**Figure 2:**
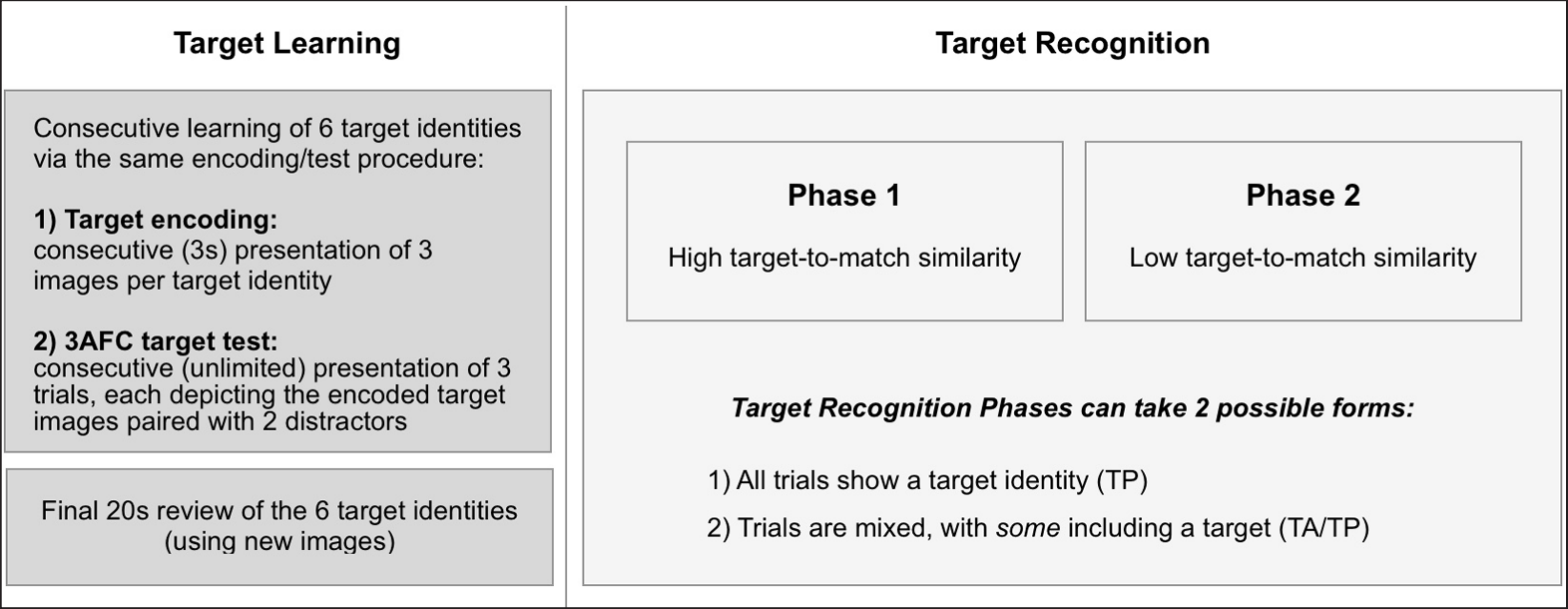
**Experimental design**. The experiment starts with *Target Learning* followed by *Target Recognition*. During *Target Learning*, observers sequentially encode three images of a given target identity, followed by a 3-alternative forced-choice (3AFC) target test of the encoded images. Target learning of all six target identities finishes with a final 20s review of target identities using novel images. *Target Recognition* comprises two phases, which differ in their *Target-to-Match Similarity* (Phase 1: high; Phase 2: low; see Methods). *Target Recognition* Phases can differ in terms of *Target Prevalence*, i.e., they can either contain only trials depicting targets (*Target-Present;* TP), or mixed trials (*Target-Absent/Target-Present*; TA/TP). Our three groups (see Table 1) were subjected to different experimental *Contexts*, which represent our possible combinations of *Target Prevalence* (TP; TA/TP), across Target Recognition Phases with fixed order of *Target-to-Match Similarity* (low, followed by high).

To assess the effect of target-absent trials on face recognition, we created two modified versions (Group 1, Group 2; see Table 1) of the original MMT (Bate et al., 2018). At base, all versions contain two phases schematically represented in Figure 2: (1) Target Learning (consistent across versions) and (2) Target Recognition (differing across test versions). Target Recognition consists of two phases (45 trials each), which differ in terms of *Target-to-Match Similarity*. Similarity between learned targets and to-be-matched probes is higher in Target Recognition Phase 1 (“easy” trials), compared to Target Recognition Phase 2 (“difficult” trials), where paraphernalia and external facial information differ between target and probe images. *Target-To-Match Similarity* differed in the same manner across phases for all groups as described above.

Stimulus aspects aside, the three MMT versions differ in terms of *Target Prevalence* across trials, with fixed order of *Target-to-Match Similarity* (low, followed by high). In the original MMT, both Target Recognition Phases include equal numbers of target-present (TP) and target-absent (TA) trials; its setup is therefore referred to as *TA/TP—TA/TP* (see Table 1). This “original” version of the MMT was delivered to Group 3 as described above. Our two modified MMT versions both involved the same response modalities and comprised the same number of TP trials as the original. However, they differed in terms of whether the “easy” and “difficult” Target Recognition Phases contained TA trials.

For Group 1, the (easier) Target Recognition Phase 1 was identical to the original MMT (containing TA and TP trials), while the (more difficult) Target Recognition Phase 2 involved only TP trials (with a doubled number to ensure equal number of trials across phases/blocks). Group 1 is therefore referred to as *TA/TP—TP*. For Group 2 on the other hand, the experiment is structured as the opposite as Group 1, with a *TP—TA/TP* structure: its Target Recognition Phase 1 contained only TP trials (but doubled compared to the MMT), followed by the original MMT Target Recognition Phase 2.

To summarize, across Target Recognition Phases the three test versions used (Groups 1–3) contain the same (decreasing) *Target-to-Match Similarity* (high; low), with varied *Target Prevalence* (TA/TP; TP). For all versions, observers provided their responses by button press, indicating whether any of the probes matches a target identity (by pressing 1, 2 or 3), or not (by pressing 0). They were aware of the type of manipulation (High or Low *Target-to-Match Similarity*/High or Low *Target Prevalence*) before each Testing Phase. When a phase contained TP trials only (High *Target Prevalence*), observers could not respond by pressing button 0, only buttons 1,2 or 3 could validate a response and pass to the next stimuli.

## Statistical Analyses

### Measures

As across MMT versions, not all Target Recognition Phases included TA trials. These versions were the result of the combination of Target Recognition Phases with different *Target Prevalence*. Therefore, to compare performance between contexts and phases (by considering *Hit Rate*, rather than *Accuracy* as the dependent variable), our analyses were conducted only on TP trials, while TA trials (*False Alarms* or *Correct Rejections*) were not considered.

### Replication of the Original MMT

First, to ensure construct validity, we compared our data obtained using the original MMT version (which is equivalent to Group 3 regarding the experimental conditions: *TA/TP—TA/TP Target Prevalence* combination) to those reported by Bate et al. (2018). To this end, we compared *Accuracy* and *Sensitivity* (d’), as well as *hit* and *correct rejection rates* between studies. Note that since, unfortunately, the original MMT’s authors could not provide their observers’ individual data, we could only compare data between studies at the mean Accuracy level via one-sample t-tests (wherein Bate et al.’s (2018) across-observer means represent μ_0_).

### Linear Mixed-Effects Modelling

To assess potential effects of *Target-to-Match Similarity, Target Prevalenc*e and *Cultural Exposure* on observers’ hit rates, we successively fitted linear mixed effects models to observer-level data (R, Version 4.0.5; R Core Team, 2013; lme4 package; Bates, Maechler, Bolker, & Walker, 2015), allowing us to compute each factor’s Bayesian Information Criterion (BIC). As a general strategy, we began by fitting *Hit Rate* data to a null model (Model 0, including an intercept term only). Subsequently, we compared it against more complex models, successively adding a single fixed effect (i.e., *Target-to-Match Similarity*, or *Target Prevalence*) to each one, then calculating the Bayes Factor (BF) between Models *n* and *n+1* (where *n* denotes the last favored—and least complex—model). We would then retain whichever model the BF favored for subsequent comparisons. In any case where multiple models of rank n+1 were equally favored over model n, the AIC was used to adjudicate between them by selecting the most parsimonious among them. Finally, we added *Cultural Exposure* (and associated interactions) to the most favored fixed-effects model as a random factor (since this was neither controlled nor assigned) in the same iterative manner.

### Context effects: Target-to-Match Similarity and Target Prevalence

The models described above tested for effects of *Target-to-Match Similarity* and *Target Prevalence* across the two modified versions of the MMT (Groups 1 and 2), and the original MMT (Group 3). To assess the influence of *Target Prevalence Context* across *Target-to-Match Similarity*, we compared each of our 3MT cohorts to our MMT cohort using the same strategy outlined above, with two more sets of linear mixed-effects models. Effectively, these tested the effects of changing *Target Prevalence* from Low to High or vice versa on performance in the *Low Target-to-Match Similarity*. In all cases, following model selection, we compared relevant marginal conditions for the significant factors with t-tests.

## Results

### Replication of the original Models Memory Test (MMT)

First, comparing the mean performance of observers who completed the original MMT (in our replication through Group 3) against results from Bate and colleagues (2018), we obtain similar results overall. Despite obtaining generally higher means for overall *Accuracy, Hit Rate, Correct Rejection Rate* as well as *Sensitivity*, t-tests comparing the samples’ means between studies yielded no significant differences (see Table 2). Additionally, we confirmed the absence of a statistical difference by calculating per measure the effect size (Cohen’s D, in Table 2) of the difference across cohorts. Overall, these results suggest that (at the group level) observers in Group 3 achieved comparable performance to the mean reported by Bate et al. (2018).

**Table 2:** Comparison of behavioral performance between the cohort reported for the original Models Memory Test (Bate et al., 2018) and our sample (Group 3).


	ORIGINAL MMT N = 40 (33Y)	GROUP 3 N = 20 (37Y)	DIFFERENCE BETWEEN MMT VERSIONS	EFFECT SIZE OF DIFFERENCE COHEN’S D

Mean ACC ± SD	.54 ± .14	.57 ± .16	t(19) = 0.72, p = .4777 > .05	.16

Hit Rate ACC ± SD	.51 ± .20	.53 ± .16	t(19) = 0.62, p = .5422 > .05	.14

Correct Rejection Rate ACC ± SD	.57 ± .23	.60 ± .21	t(19) = 0.61, p = .5474 > .05	.14

d’ ± SD	.26 ± .84	.38 ± .93	t(19) = 0.58, p = .38 > .05	.13


### Separating Effects of Target-to-Match Similarity and Target Prevalence on Hit Rate

**Figure 3:**
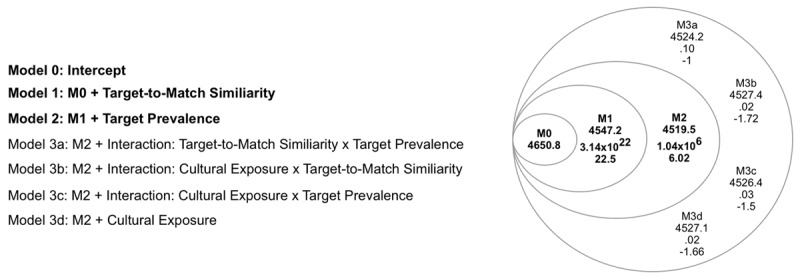
**Multi-level models per factor independently of group**. Numbers below each model name represent (in order) the Bayesian information criterion (BIC), the Bayes Factor (BF), and the BF’s logarithmic expression (Log_10_). Black font indicates models showing better evidence of explaining the variance among participants in comparison with the inferior level’s model. The highest model in black is gathered for further analyses.

We modeled the effects of hit rate as described in the Statistical Analyses section. Bayes Factors comparing the models (see Figure 3) decisively support Model 2 compared to Models 0 or 1. Thus, we retained Model 2 (including *Target-to-Match Similarity* and *Target Prevalence* as main effects on hit rate). A paired samples t-test yielded a significant difference between hit rates observed in the high (Mean = .70, SD = .20) vs. low (Mean = .53, SD = .20) *Target-to-Match Similarity* conditions (t(59) = 6.76, p < .05). As anticipated, observers’ hit rates were generally lower during Target Recognition Phase 2, thereby confirming increased task difficulty via more extreme ambient changes. An independent-samples t-test investigating the two conditions of the *Target Prevalence* yielded a significant difference between high (Mean = .71 ± .19) and low (Mean = .57 ± .21) *Target Prevalence* (t(118) = –3.55, p < .05): observers generally performed better under high *Target Prevalence* scenarios.

### Contextual Effects: Target-to-Match Similarity and Target Prevalence

**Figure 4:**
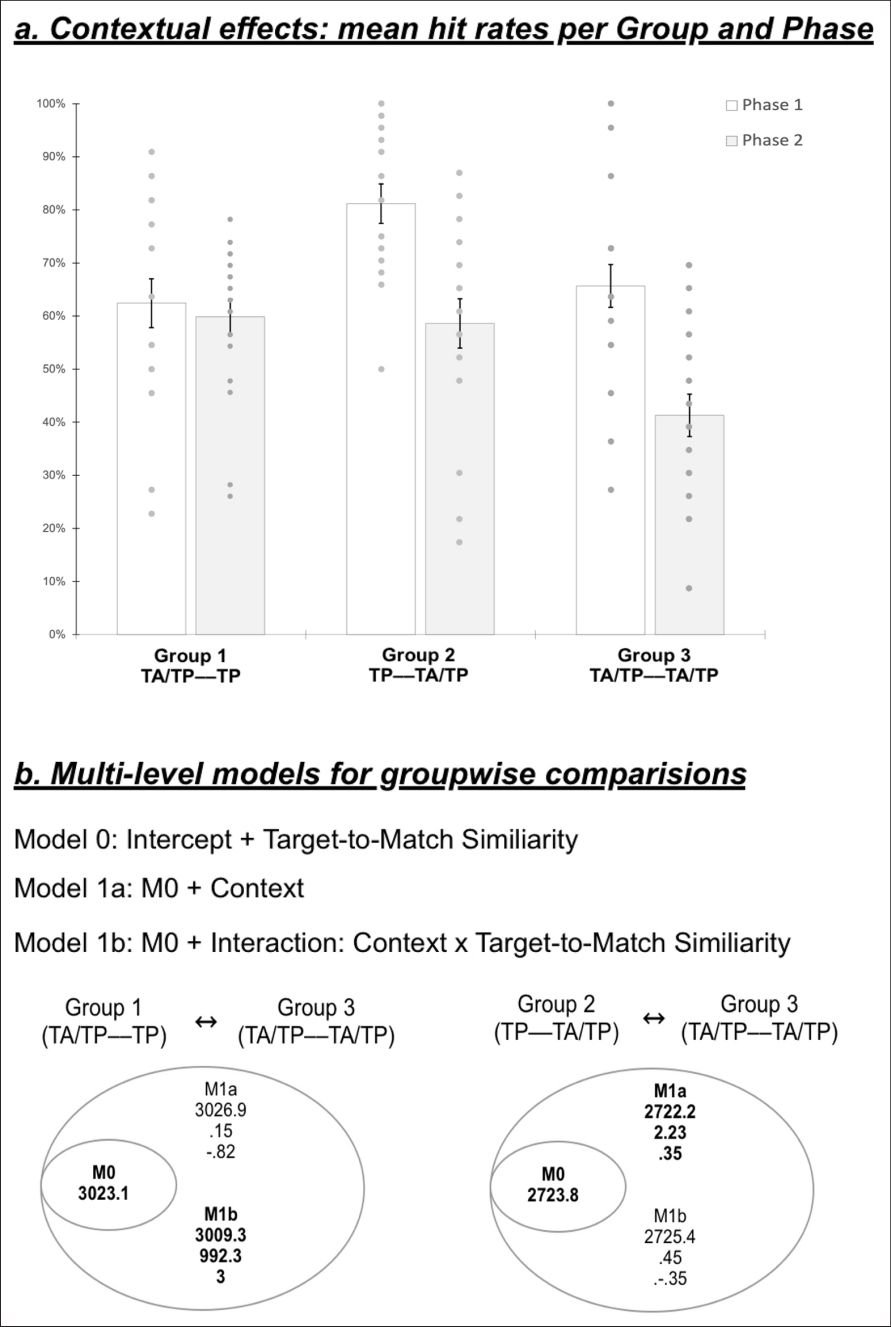
**Groups’ performance and multi-level model results. a**. Mean hit rates per group and Target Recognition Phase. Multi-level model results for **b.** Group 1 vs. Group 3, and **c.** Group 2 vs. Group 3. Numbers below each model name represent (in order) the Bayesian information criterion (BIC), the Bayes Factor (BF), and the BF’s logarithmic expression (Log_10_). Black font indicates models showing better evidence of explaining the variance among participants in comparison with the inferior level’s model. The highest model in black is gathered for further analyses.

We conducted separate model comparisons between the cohorts who completed modified versions of the MMT (Group 1 and 2), and our original MMT cohort (Group 3) (for details, see Statistical Analyses). Figure 4a displays groups’ mean hit rates for Target Recognition Phases 1 and 2 (across which *Target-to-Match Similarity* decreased); Figure 4b displays the results of the multi-level models detailed below. Specifically, here we sought to determine whether the effect of Context on hit rate depends on the presence of TA trials in Phase 1 or Phase 2.

#### For constant Phase 1 Context: Group 1 (TA/TP—TP) vs 3 (TA/TP—TA/TP)

To begin with, we considered the scenario where Phase 1 was identical between groups, changing only during Phase 2 for Group 1. Having confirmed a general effect of target similarity (Figure 3), we treated this as our zero-order model, and compared it against models including a main effect of Context (Figure 4b; model 1a) and a Context by Similarity interaction (Figure 4b; model 1b). While Model 1a provides no better explanation than Model 0, we find decisive evidence favoring Model 1b over Model 0. Overall, this suggests that the interaction between *Target Prevalence* and *Target-to-Match Similarity* best explains observers’ pattern of hit rates.

Post-hoc independent-samples t-tests revealed no difference in hit rates for Phase 1 (*High Target-to-Match Similarity*; t(37) = –.53, p > .05; M1 = .62 ± .20 vs. M3 = .66 ± .18) when Groups initially experienced the same initial conditions (target absent on half of trials). However, in Phase 2 (*Low Target-to-Match Similarity)*, we find significantly higher hit rates for Group 1 (M = .60, SD = .14) than Group 3 (M = .41 ± .18), (t(37) = 3.56, p < .05). Overall, the observed interaction effect suggests that completing Phase 1 including TA trials differentially influenced Phase 2 Hit Rate, such that the change in context (i.e. when only TP trials were shown in Phase 2) influenced Hit Rate in Phase 2.

#### Phase 1 Context varied between Groups: Group 2 (TP—TA/TP) vs 3 (TA/TP—TA/TP)

Next, we sought to examine whether the effect of Similarity also depended on Context: would hit rates in fact decrease following exposure to low (versus high) target prevalence during Phase 1. The Bayes Factor between models including versus excluding Context as a factor (either alone or interactively, while accounting for Similarity) favored neither one. However, comparison of AIC between models suggests that the model including only the *Context* main effect is the most parsimonious (ΔAIC = –7.3). Within Model 1aA, we find a main effect of *Target Prevalence Context*. A post-hoc independent samples t-test of this effect revealed that observers assigned to Group 2 performed significantly better than those in Group 3 ((t(80) = 3.39, p < .01); M2 = .70 ± .22 vs. M3 = .54 ± .216; t(80) = 3.39, p < .05).

## Discussion

Our systematic investigation of *Target Prevalence, Target-to-Match Similarity, and Context* as factors influencing neurotypical face recognition performance finds that TA trial prevalence influences observers’ hit rates regardless of the levels of other factors. Overall, their inclusion in MMT-type tasks deteriorates observers’ hit rates, which ought to be expected given the results of visual search studies for non-face stimuli (Wolfe & Van Wert, 2010; Wolfe et al., 2007). Practically speaking, our results suggest that, while inclusion of TA trials are important in gauging sensitivity in FIP measures, these are likely prone to intra-observer bias. As such, care should be taken to interpret performance measures while either controlling for TA trial prevalence, or systematically varying it.

Aside from that, we did not find any interaction with *Target Prevalence*. As discussed below, this suggests that response bias is unaffected by other factors that can cause variation in hit rate. This is obviated by considering the effect of eliminating TA trials on response bias: observers’ decisions are then *forced* to be between target locations or response keys (not between signal and noise), thus any residual bias no longer corresponds to a shift in criterion (preference for reporting signal or noise), but purely in preference for one or another response button, or image location. Consequently, studies including TA trials underestimate hit rates, and likely do not (or cannot) control for this by any other manipulation (e.g., similarity between learned targets and probe stimuli), since these effects are separable. Rather, systematic variation or control over target prevalence are necessary to minimize criterion changes.

### Target-to-Match Similarity

One of our goals was to investigate the effect of similarity between an encoded target identity and its matching probe items in a modified MMT. We find increased hit rates between Target Recognition Phases 1 and 2 (which shift from high to low *Target-to-Match Similarity*), closely replicating Bate and colleagues’ (2018) originally reported results. The conditions experienced by Group 1 of our study tightly mirror those of the original MMT, so it seems unlikely that the between-group *Context* effects we find here are attributable to methodological differences between studies.

We further find strong evidence for an effect of *Target-To-Match Similarity*; participants’ performance was better in the first Target Recognition Phase, where similarity was higher compared to the second one. This aligns with previous studies suggesting that more pronounced changes in the appearance of recently learned target identities negatively affect recognition (Ellis, 1975; Patterson & Baddeley, 1977). Note, however, that the MMT’s experimental design (Bate et al., 2018) involved a fixed order, i.e., higher followed by lower similarity across Target Recognition Phases (vs. a potentially fully randomized trials order with respect to their Target-to-Match Similarity).

### Target Prevalence

Previous studies investigating the effect of *Target Prevalence* outside the domain of face processing have reliably shown that the frequency of target (or signal) occurrence strongly influences response bias, such that reporting of signals is commensurate with their prevalence (Wolfe & Van Wert, 2010; Wolfe et al., 2007). We sought to determine the impact of TA trials on face recognition performance by modifying the MMT. Its Target Recognition Phases involve only TP trials, so we devised versions including equal proportions of TP and TA trials in either or both Phases. Mirroring previous findings from the visual search literature mentioned above, we observe reduced hit rates when TP trials are embedded among TA trials.

Overall, we believe that our findings concerning the negative impact of low *Target Prevalence* on hit rates during face recognition extends to other FIP reliant (including applied) scenarios, we anticipate observing inter-individual differences in the expression of this effect. Once confirmed, this would support our view that task-specific training (e.g., of radiologists, luggage screeners, law enforcement professionals) should include a combination of TP and TA trials, as well as characterize the effect of target prevalence variations on individuals’ performance. In addition to approximating real-world conditions, this could aid observers in guarding against the decision biases they express during recognition tasks, and ideally reduce their impact in applied settings.

### Context effects

Wolfe and colleagues (2007) have previously reported that visual search performance varies with the frequency of a target’s occurrence and position. Specifically, initial training with high (vs. low) *Target Prevalence* affected performance in a subsequent low prevalence phase. Our final aim was to characterize this contextual carryover effect, in the context of face recognition memory.

First, we found that Group 1 (TA/TP—TP) achieved higher Hit Rates than Group 3 (TA/TP—TA/TP; original MMT) on average. Having been exposed to the identical procedure in the first Target Recognition Phase (with low *Target Prevalence*), the only between-groups factor that could explain this performance difference is *Target Prevalence* in the second Target Recognition Phase, characterized by lower *Target-to-Match Similarity*. While Group 3 experienced low *Target Prevalence* throughout both phases, Group 1 only experienced high *Target Prevalence* during the second Target Recognition Phase. The effect of *Target Prevalence* between Groups 1 and 3 suggests that high *Target Prevalence* counteracts the increased difficulty due to lower *Target-to-Match Similarity* across phases.

Second, to investigate a potential contextual effect, we compared the performance of Group 2 (TP—TA/TP) and Group 3 (TA/TP—TA/TP). Unlike the previous comparison, the favored model accounted for only the Context, but not the interaction between Context and Target-to-Match Similarity. This was explained by a main effect of Context, due to Group 2 exhibiting significantly better performance than Group 3. If Group 2’s observers had highest *Hit Rates* for both phases compared to those from Group 3, we cannot account for any specific contextual effects regarding *Phase 1* or *Phase 2*. This is because of the relative non-significance of the model 1b including the interaction between *Context* and *Target-to-Match Similarity* (Figure 4b; model 1b). Consequently, we can only talk about a general contextual effect between both groups on the Hit Rate.

Here, in line with our expectation, and similar to the aforementioned findings (Wolfe et al., 2007), *Target Prevalence* in the first Target Recognition Phase affected performance in the second phase. We observed a behavioral advantage for initial high (TP) vs. low (TA/TP) prevalence, with the prior leading to better performance at a second low prevalence (TA/TP) phase. Thus, when varying *Target Prevalence* dichotomously, we observed a systematic response bias related to the target’s occurrence.

### Limitations

The present study was designed to explore factors potentially affecting face recognition performance in the MMT (Bate et al., 2018), which was recently introduced as a more ecological alternative to the well-established CFMT+ (Russell et al., 2009). While our observations support the notion of important contextually determined biases, these findings arose in the context of a restricted set of experimental conditions.

First, as mentioned previously, we did not implement all possible contexts, thereby lacking the TP–TP condition. Second, following the original MMT design, across Target Recognition Phases, *Target-to-Match Similarity* always decreased (high followed by low); the opposite direction was never tested. A complete experimental design involving all possible combinations would entail four different contexts (TP—TP, TP—TA/TP, TA/TP—TP and TA/TP—TA/TP) as well as counterbalanced orders of the *Target-to-Match Similarity* and the *Target Prevalence*. This would preclude the possibility of a cohort or confounding effect. Third and finally, we treated *Target Prevalence* dichotomously, and further studies are needed that systematically vary the ratio of TP:TA trails within TA/TP contexts. *Target Prevalence* effects in FIP and other visual search tasks are relatively ubiquitous and domain-general. To determine why target recognition performance is negatively affected by low *Target Prevalence*, Wolfe and colleagues’ (Wolfe & Van Wert, 2010; Wolfe et al., 2007) analyzed signal sensitivity (d’), as well as response times for visual search tasks. They concluded that prevalence influences decision criterion and, therefore, the perceptual decisions about an item.

Our findings were obtained in the context of a recognition task, which is analogous to a 3 items visual search. As such, the presently observed effects would be likely to differ in tasks where simultaneous matching is probed, i.e., those devoid of a memory component. Of note, a learning effect across the trials cannot be excluded. Indeed, observers assigned to Group 2 were exposed to 45 TP trials (high *Target Prevalence*) during *Phase 1*. As such they had roughly twice the amount of exposure to target identities as compared to observers in Group 3 (exposed to TA/TP trials in the low *Target Prevalence Phase 1*). To eliminate such a potential effect, a more complex design would be required to ensure comparable TP exposure.

Finally, our analyses focused on the only metric comparable given the between-group variation in *Target Prevalence* across Target Recognition Phases: hit rate. Future studies could include an extended experimental design, via introduction of a fourth *TP––TP* context, in combination with a within-observer approach, whereby participants complete different contexts (e.g. TP—TA/TP and TA/TP—TP), and sensitivity analyses.

## Conclusion

Inspired by visual search studies reporting variations in decisional biases and error rates related to varied *Target Prevalence*, the present study sought to address generally acknowledged, but empirically under-investigated factors assumed to influence face recognition performance: *Target Prevalence, Target-to-Match Similarity* and contextual effects. We reasoned that all are crucial across various applied visual tasks, including radiology, baggage screening, and suspect identification in law enforcement. Our findings suggest that all three factors influence visual recognition performance, but not necessarily interactively. This has general implications for test development and training of professionals performing visual tasks in more realistic situations. With the mentioned factors, we propose means in which this work could be extended to allow a more fine-grained investigation of the reported effects, including an individual differences approach.

## Data Accessibility Statement

All data and code can be found on the accompanying OSF project for this publication (https://osf.io/3swhx/).

## Abbreviations

Face identity processing (FIP); Artificial intelligence (AI); Target present (TP); Target absent (TA).
